# Noninvasive imaging techniques for the diagnosis of cutaneous larva migrans

**DOI:** 10.1111/srt.13126

**Published:** 2021-12-19

**Authors:** Marco Campoli, Giulio Cortonesi, Linda Tognetti, Pietro Rubegni, Elisa Cinotti

**Affiliations:** ^1^ Dermatology Section Department of Medical Surgical and Neurological Sciences Santa Maria Alle Scotte Hospital Siena Italy; ^2^ Department of Medical Biotechnologies University of Siena Siena Italy

**Keywords:** imaging, larva, migrans, noninvasive

**FIGURE 1:**
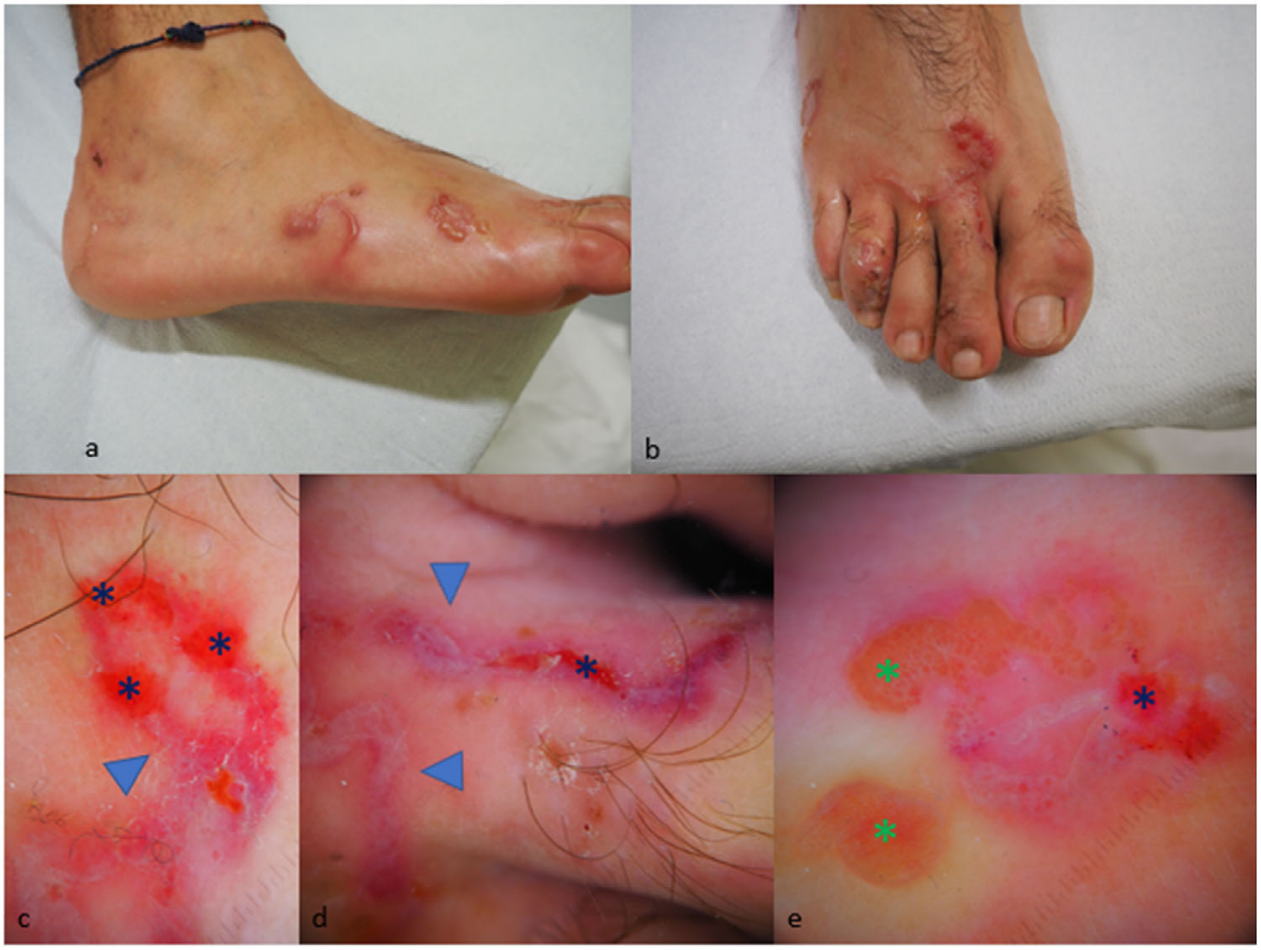
Clinical images (A, B) and dermoscopy (C–E) that shows erythematous linear track (blue triangles) with some erosions (blue asterisks) and yellowish vesicles (green asterisks)

**FIGURE 2:**
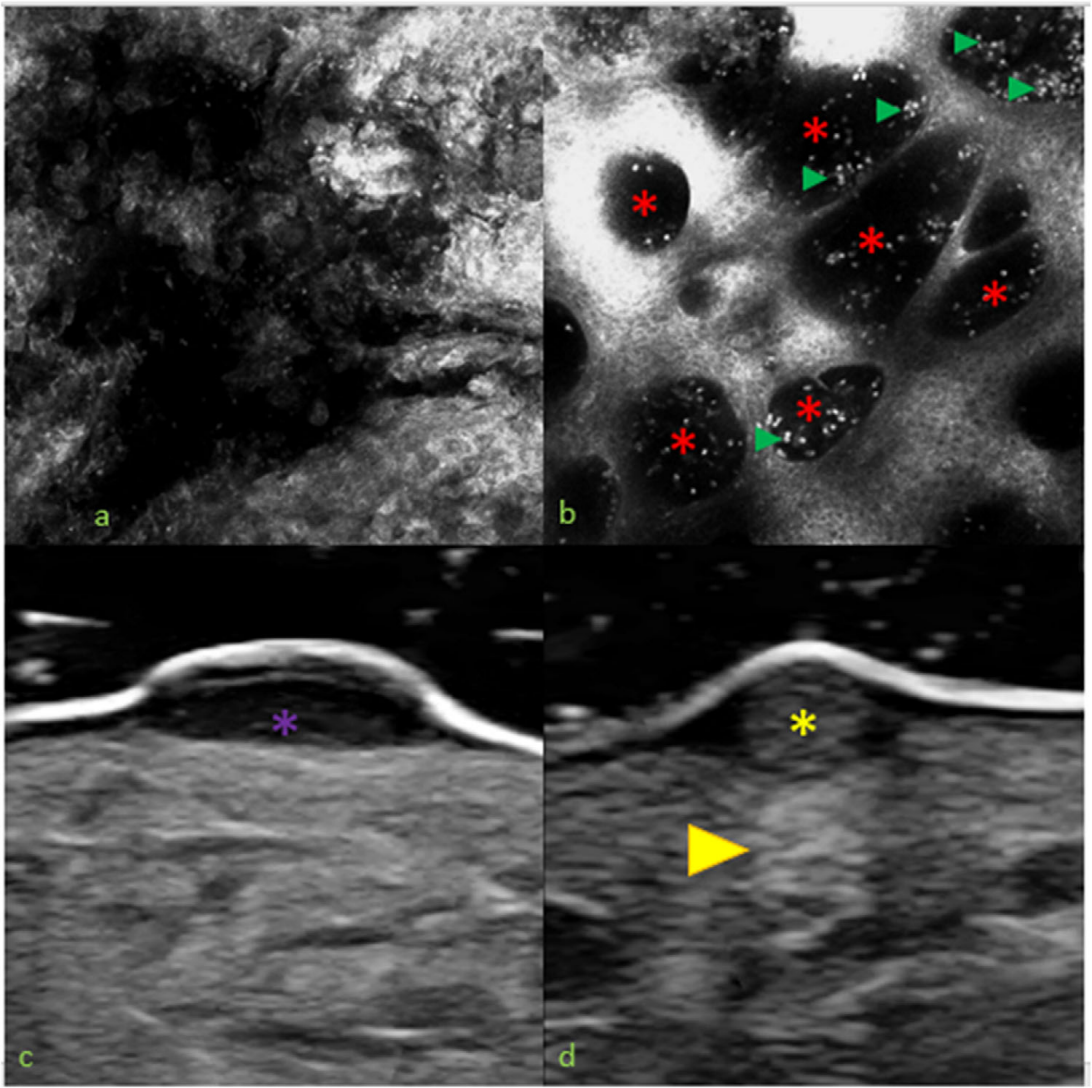
Reflectance confocal microscopy (RCM) (A, B) and high frequency ultrasound (HFUS) images (C, D). RCM shows a dark disruption in the normal honeycomb pattern of the epidermis corresponding to the larval burrow (A) and dark oval structures corresponding to vesicles (B, red asterisks) and many small hyperreflective point structures corresponding to inflammatory cells (B, green arrows). HFUS revels anechoic cylindrical structure corresponding to the larval burrow (C, violet asterisks) and homogeneoulsy hyperechoic cylindric mass (D, yellow asterisks) generating a posterior echo (yellow arrow), which could correspond to the parasite

A 31‐year‐old man presented with a 3‐week history of a migratory, itching rash with blisters on his right foot. He reported walking barefoot on the sand during a trip to the Brazilian coasts some weeks before. Physical examination showed serpiginous erythematous papules on the foot with bullous lesions and serum oozing erosions suggestive of cutaneous larva migrans (CLM) (Figure 1). Dermoscopy revealed erythematous linear track with some erosions and yellowish vesicles. Reflectance confocal microscopy (RCM, VivaScope 3000, Caliber, USA) confirmed the presence of a hyporeflective disruption in the normal honeycomb pattern of the epidermis corresponding to the larval burrow (Figure 2A and B). In some areas, it was possible to observe hyporeflective oval areas corresponding to vesicles with many small hyperreflective point structures corresponding to inflammatory cells. High‐frequency US (HFUS) 20 MHz showed anechogenic bulla with moderately hypoechoic structures inside, probably corresponding to inflammatory infiltrate (Figure 2C and D). At the terminal part of the burrow, we found a homogenously hyperechoic cylindrical structure, probably corresponding to the parasite; the slightly hyperechoic roundish echo generated from the mass was likely to be parenchymatous in consistence. Our patient was treated with oral albendazole 400 mg for 3 days with significant improvement of symptoms within few days and complete resolution of the lesions within two weeks.

CLM is a cutaneous infestation by nematode larvae, particularly *Ancylostoma braziliense* and *Ancylostoma caninum* animal hookworms.^1^ These parasites usually live in animal intestines, where their eggs passed in feces of cat or dog host.^1^ It commonly occurs from direct skin contact to contaminated soil or sand, which contains these worms in tropical and subtropical areas.^1^ This disease is characterized by a pruritic, erythematous, linear, or serpiginous, migratory track, mainly on the lower extremities. The larvae are unable to migrate through the basal membrane because of deficiencies in the collagenase enzyme.^2^ CLM heals spontaneously within a few weeks to months and anthelmintic therapies can shorten the duration of the disease.^3^ Exaggerated reactions to the worm, such as bullous lesions found in our case, are not very frequent and there are only few cases described in the literature.^3^, ^4^, ^5^, ^6^

New noninvasive diagnostic techniques are increasingly used in dermatological clinical practice.^7^ To our knowledge, there are no cases concerning the use of ultrasounds in patients affected by CLM and there is only one RCM report.^8^ In this case, the authors described the presence of a dark disruption of the epidermis and a highly refractile oval structure that they supposed to be the larva.^8^ However, the image provided by the article seems to correspond more to an acrosyringium that characterizes acral skin than to a larva.^9^, ^10^ This fact highlights the difficulty of identifying and describing for the first‐time new features with novel imaging techniques.

In our case, HFUS seemed to provide direct images of the parasite within the patient's skin; RCM was not able to detect the parasite but gave indirect signs of its presence. Besides revealing the disarrangement of the epidermis, RCM highlighted the presence of vesicles and numerous inflammatory cells corresponding to the exaggerated inflammatory response of the host, which led to the formation of blisters. Although the diagnosis of CLM remains clinical, RCM and HFUS are new noninvasive imaging techniques that could provide additional information to support the diagnosis in clinically atypical cases, as in other cutaneous infestations.^11^ Further studies are needed to confirm our observations.
